# Supplementary material for: Mitochondrial Peroxiredoxin 3 Is Rapidly Oxidized and Hyperoxidized by Fatty Acid Hydroperoxides
Source: Antioxidants (Basel). 2023 Feb 7;12(2):408. doi: 10.3390/antiox12020408 (PMC9952270; doi:10.3390/antiox12020408)
Supplement: Supplementary file 1 [file antioxidants-12-00408-s001.zip › antioxidants-2154269-supplementary.pdf]

Supplementary Materials

A) B) C) D) E)

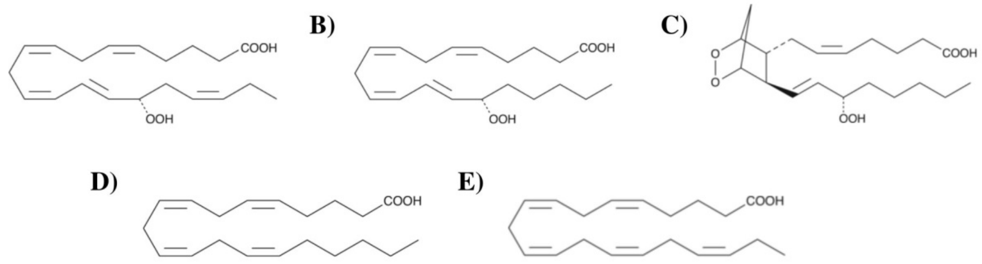

Supplementary Figure S1. Molecular representations of $_{f}$FA-OOH and FA used throughout this work. (**A**) 15S-hydroperoxy-5Z,8Z,11Z,13E,17Z-eicosapentaenoic acid (15(S)-HpEPE); (**B**) 15S-hydroperoxy-5Z,8Z,11Z,13E-eicosatetraenoic acid (15(S)-HpETE); (**C**) 9α,11α-epidioxy-15S-hydroperoxy-prosta-5Z,13E-dien-1-oic acid (PGG_2); (**D**) arachidonic acid (AA) and (**E**) eicosapentaenoic acid (EPA).

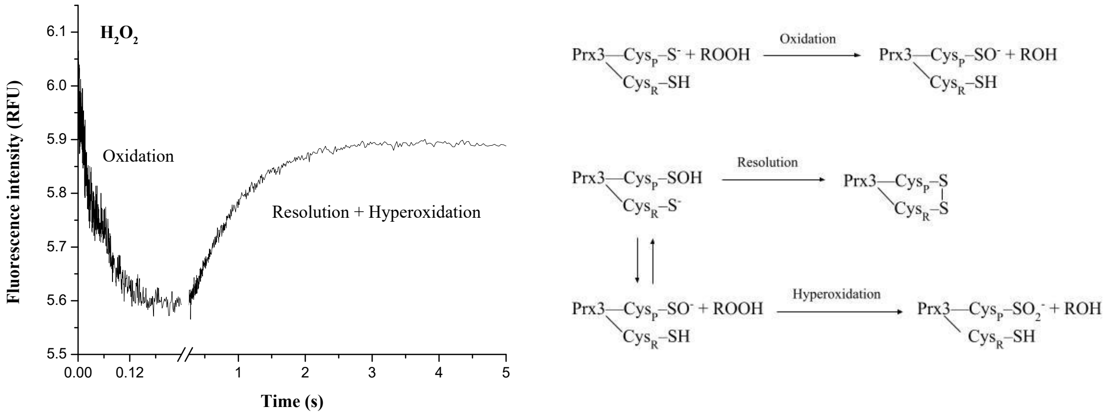

Supplementary Figure S2. Intrinsic fluorescence changes in *Hs*Prx3 caused by Cys_P oxidation, resolution and hyperoxidation. Oxidation (from thiol to sulfenic acid) and hyperoxidation (from sulfenic acid to sulfinic acid) in *Hs*Prx3 are bimolecular processes while resolution is intramolecular and depends on a change from the so-called fully folded to a locally unfolded conformation followed by the condensation reaction between the sulfenic acid in Cys_P and the thiolate in Cys_R from another protein subunit. In the case of reaction with H_2O_2, in which the rate constant of hyperoxidation is 1.1 x 10^3 $M^{-1}s^{-1}$, hyperoxidation does not compete with resolution (2 s^{-1} at pH 7.8 and 14ºC) at <50 µM oxidant concentration. Adapted from [13].

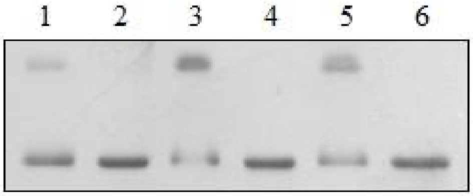

Supplementary Figure S3. Effect of 15(S)-HpETE *versus* H_2O_2 on the distribution of *Hs*Prx3 between monomeric and disulfide-bonded dimeric species. SDS-PAGE of reduced *Hs*Prx3 (5 µM) without treatment (lane 1 w/o β-ME, lane 2 with β-ME); exposed to H_2O_2 (500 µM) (lane 3 w/o β-ME, lane 4 with β-ME); exposed to 15(S)-HpETE (10 µM) (lane 5 w/o β-ME, lane 6 with β-ME).

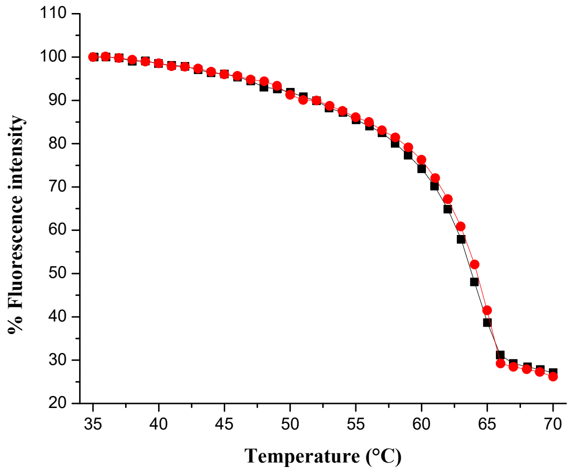

Supplementary Figure S4. Effect of ethanol on the heat-induced unfolding of *Hs*Prx3. Heat unfolding transitions following the intrinsic fluorescence intensity (λ_{ex} = 295 nm, λ_{em} = 335 nm) of the tryptophans of *Hs*Prx3 (4 μM) in the absence (■) and presence of the same volume of ethanol (●) that was used to solubilize AA.

```
VTQHAPYFKGTAVVNGEFKDLSLDDFKGKYLVLFFYPLDFTFVCPTEIVAFSDKANEFHD
+ + AP FK TAVV+G FK++ L D+KGKY+VLFFYPLDFTFVCPTEI+AFS++A +F
IGKPAPDFKATAVVDGAFKEVKLSDYKGKYVVLFFYPLDFTFVCPTEIIAFSNRAEDFRK

VNCEVVAVSVDSHFSHLAWINTPRKNGGLGHMNIALLSDLTKQISRDYGVLLEGSGLALR
+ CEV+ VSVDS F+HLAWINTPRK GGLG +NI LL+D+T+++S DYGVL    G+A R
LGCEVLGVSVDSQFTHLAWINTPRKEGGLGPLNIPLLADVTRRLSEDYGVLKTDEGIAYR

GLFIIDPNGVIKHLSVNDLPVGRSVEETLRLVKAFQYVETHGEVCPANWTPDSPTIKPSP
GLFIID  GV++ ++VNDLPVGRSV+E LRLV+AFQY + HGEVCPA W P S TIKP+
GLFIIDGKGVLRQITVNDLPVGRSVDEALRLVQAFQYTDEHGEVCPAGWKPGSDTIKPNV

AASKEYFQKVN
  SKEYF K N
DDSKEYFSKHN
```

Supplementary Figure S5. Sequence alignment of mature *Hs*Prx3 (black) and *Hs*Prx2 (blue). Residues that make up the hydrophobic patch near Cys_P in *Hs*Prx3 are conserved in *Hs*Prx2 (indicated in yellow).
